# Reproducibility of a Digital Method to Evaluate Soft Tissue Modifications: A study of Inter and Intra-Operative Measurement Concordance

**DOI:** 10.2174/1874210601711010171

**Published:** 2017-03-31

**Authors:** Davide Rancitelli, Marco Cicciù, Francesco Lini, Davide Fumagalli, Anna Chiara Frigo, Carlo Maiorana

**Affiliations:** 1 Department of Dental Implants, Maxillofacial Surgery and Odontostomatology Unit, University of Milan, Milan, Italy; 2 Department of Biomedical and Dental Sciences and Morphofunctional Imaging, University of Messina, Messina, Italy; 3 Department of Cardiological Sciences, University of Padua, Padua, Italy

**Keywords:** Periodontal probe, Soft tissues, Periodontal disease, Peri-implant tissues, Apico-coronal migration

## Abstract

**Background:**

The soft tissue healing and quality around dental implant is a current debated topic in the recent literature. The free gingival margin and the quantity and quality of the mucosa around teeth and surrounding dental implants still determine the hard and soft tissue healing status.

**Objective:**

The aim of this study is to assess inter and intra-operative measurement concordance of a method aimed at evaluating the apico-coronal migration of free gingival margin, using intra-oral photography.

**Methods:**

The method was tested on peri-implant tissues that were treated with a connective tissue graft on the second stage surgery. Thirty-eight measurements on 13 implants in 8 patients were recorded. An intra-oral photograph was taken for the graft and the provisional crown, which enclosed a circular landmark with a previously determined diameter. The landmark was prepared with a red-resin by the same technician. Before crown cementation, the landmark was calibrated with a digital calibrator by the main investigator, to determine the precise diameter up to two decimal numbers.

**Results:**

On the intra-oral photograph, the distance was measured from the most apical point of the determined landmark to the zenith of the gingiva, using an image-processing program designed for scientific multidimensional images (Image J). Three independent examiners took these measurements.

**Conclusion:**

The main advantage of the present non-invasive technique is that the spatial plane of the prosthetic landmarks is the same of the tooth unlike the utilization of periodontal probe, which is generally positioned on different plans in the space (generally more vestibular).

## INTRODUCTION

The monitoring of soft tissue changes is an essential factor to assess short and long term results of prosthetic, periodontal and implant therapy [1, 2]. The most recent classifications of periodontal diseases published by the consequence of Consensus Conference about periodontal disease and case definitions [3, 4] were based on measurements of attachment level, probing depth, bone loss and/or degree of inflammation. Health (or “no disease”) was defined as concurrent absence of these signs and symptoms and was always related to such measurement and mm values.

Periodontal health is a clinical condition related to the absence of progressive attachment loss around the tooth. Several recent findings suggest that the lack of consensus in the definitions of periodontal disease may be connected with the difficulties on having conformity about diagnosis by the clinicians. However, the combination of X-ray investigation and individual clinical outcome parameters, such as periodontal deep (PD), bleeding on probing (BOP), and others, provide the basis to assess classification systems for oral health and periodontitis, and they provide the basis to improve diagnosis accuracy [1-6]. The periodontal probe is mostly used in common clinical practice to perform this type of measurements like keratinized tissue amount, papilla height, and gingival recessions with a mean error of 1 mm [3-6]. This imprecision can generate an incorrect interpretation by the clinician, which can potentially result in an alteration of the data reported in several studies comparing different therapeutic treatments or techniques. In the recent literature, several digital techniques have been proposed to avoid this problem and to improve the precision of the evaluation methods. Some protocols use measurements on virtual models obtained by optical scans taken intraorally [7-10]. Others instead, exploit radiodense marker [9] or prosthetic landmarks to perform measurements using digital technology, respectively, with cone beam CT or intra-oral photographs [11-13]. The common feature to these approaches is the possibility to perform repeatable and comparable measurements with accuracy greater than periodontal probes [14]. It would seem that digital measurements improve the reliability and most of all the reproducibility of the measurements.

The aim of the present study was to assess inter and intra-operative concordance of a measurement method aimed at evaluating the apico-coronal migration of the free gingival margin, using intra-oral photography combined with a digital software dedicated for scientific measurements.

## MATERIALS AND METHODS

The present investigation was performed accordingly with the declaration of Helsinki rules.

The method was tested on peri-implant tissues that received a connective tissue graft on the second stage surgery. Peri-implant tissues were assessed up to four follow-up appointments (30, 60, 90, 120 days after the provisionalization of the implants). Thirty-eight measurements on 13 implants (Global, Sweden & Martina, Padua, Italy^®^) in 8 patients were recorded Figs. (**1a**, **2a**). In every follow-up appointment, an intra-oral photograph was taken for the graft and the provisional cemented crown, which enclosed a circular landmark, centered in the middle of the tooth, with a previously determined diameter. The landmark was prepared with a red-resin by the same technician Fig. (**3**). Before crown cementation, the landmark was calibrated with a digital hi-precision caliper (Thomas Scientific Swedesboro, USA) by the main investigator, to determine the precise diameter up to two decimal numbers Fig. (**4**).

Considering the second examiner (F.L), the Bland-Altman analysis indicates that the 95% limits of agreement between the mean of the two readings made in different times ranged from -0.76 to 0.43 mm. The medium difference was 0.16 mm.

Considering the third examiner, the Bland-Altman analysis indicates that the 95% limits of agreement between the two readings ranged from -0.35 to 0.27 mm. The medium difference was -0.037 mm.

Considering the inter-operators concordance, the Bland-Altman analysis indicates that the 95% limits of agreement between the two mean readings ranged from -0.29 to 0.54 mm. The medium difference was 0.124 mm

Considering the inter-operators concordance, the Bland-Altman analysis indicates that the 95% limits of agreement between the mean two readings ranged from -0.35 to 0.28 mm. The medium difference was 0.048 mm.

On the intra-oral photograph, the distance was measured from the most apical point of the determined landmark to the zenith of the gingiva, using an image processing program designed for scientific multi-dimensional images (IMAGEJ 1.43u, NIH, Bethesda, MD, USA) Figs. (**5**, **6**). The same clinician, who took also the intra-oral photographs, performed surgical and prosthetic procedures. Three independent examiners who repeated the same measurements after one month in order to test inter and intra-operator concordance took the measurements with the software. The examiners were blinded about the patients’ identification and the time of the follow-up.

Image J is an open-source software ideal for scientific measurements because it can be freely inspected, modified, and redistributed. Standardized clinical intra-oral photographs were taken with a digital Single Lens Reflex camera (Nikon D90, Nikon Corp, Shinjuku, Japan) at the moment of interim crown cementation (baseline) and 1 to 4 follow-up visits to evaluate the soft tissue changes Fig. (**2a**). Every picture was acquired at 90° to the axis of the tooth using dedicated intraoral mirrors (PhotoMed International Van Nuys, Canada).

At each evaluation, the prosthetic landmark has been calibrated with the software in order to transform the pixel of the photograph in millimeters. The calibration of the landmark has permitted to delete the error caused by the inclination of the (+/- 90°) of the intraoral photography. In fact, landmark calibration at each evaluation session avoided the distortions caused by the not perpendicular position of the camera respect to the long axis of the tooth.

### Statistical Analysis

The measurements were entered into an Excel spreadsheet (Microsoft, Redmond, WA, USA) for the statistical analysis. Data were analyzed by the means of SAS 9.4 (SAS Institute, Inc., Cary, NC, USA) for Windows 10.

To evaluate the reproducibility of the method inter and intra-rater concordance was evaluated with Bland and Altman plots and Lin’s concordance correlation coefficients^12^, and 95% confidence interval (95% CI) calculated with the bootstrap method using 2000 resampling. In estimating inter-rater agreement, the mean of the two readings made in different times was considered.

## RESULTS

Results showed that the intra-rater,(CCC) was higher than 0.9 for each of the three examiners.

Inter-rater agreement was also high (CCC = 0.964, 95% CI: 0.949 - 0.980) (Table **1**).

Figs. (**1a**, **1b**, **1c** and **2a**, **2b**, **2c**) with the Bland-Altman plots illustrates the intra- and inter-rater agreement expressed as mean error and the lower (LLA) and upper (ULA) 95% limits of agreement.

Considering the first examiner (D.F) the Bland-Altman analysis indicates that the 95% limits of agreement between the two readings ranged from -0.156 to 0.188 mm. The medium difference was 0.0158 mm.

Considering the inter-operators concordance the Bland-Altman analysis indicates that the 95% limits of agreement between the two mean readings ranged from -0.47 to 0.81 mm. The medium difference was 0.172 mm.

## DISCUSSION

This study was conducted to evaluate the reproducibility of the previously described method for soft tissues changes monitoring, both between different and among the same operators. The rationale of the present research is related to a current debated topic; to find a predictable method in order to standardize and quantify the periodontal tissue consistency without measurements errors. For this reason the present study aim is to investigate by digital evaluation the consistence and the health of surrounding dental implant gingiva [1, 7, 15, 16].

The results of the investigation have shown a higher accuracy of digital measurements of intraoral measurements [5, 8] comparing the obtained data to those presented in literature. In fact, intra-operators mean errors between first and second reading were respectively 0.015, - 0.165, - 0.037 mm.

Although direct intraoral measurements have the advantage of low infrastructural requirements, they are limited by several factors. First, limited visual access to the reference points may be present. Reading errors based on different visual angles and projections of the scale of the instrument are also possible. Moreover, values are usually rounded to the next millimeter [7, 17].

The method investigated in this work consistently provide similar measures with a level of disagreement that doesn’t includes clinically important discrepancies and these findings are in line with those present in literature for digitized cast models and digital models from intraoral scanning showing inter and intra concordance of 0.99 [8, 18, 19].

Several digital methods have been recently used in medicine for diagnosis, planning, therapy, intra operative and postoperative complications management. The advantage of using digital method is related to the reducing clinical and postoperative patients’ discomfort. Moreover, a computer aided planning of a treatment seems to be useful in order to prevent all the clinical intraoperative complications [20-22].

Therefore, in this investigation, the advantage of using photographs, instead of direct clinical measurements, is a wider vision field, and it can be performed in a non-clinical environment without time pressure; they can be repeated several times if necessary and with the use of various tools possibly not suitable for intraoral applications.

Moreover, the innovative advantage of the present non-invasive approach is that the spatial plane of the prosthetic landmarks is the same of the tooth unlike the utilization of periodontal probe, which is generally positioned on different plans in the space (generally more vestibular). As a matter of principle, this could provoke a variable enlargement of the probe in the picture with the consequent problem of the digital software calibration. The integration of the red dot inside the interim crown has permitted to avoid errors caused by the projection and prospective. It’s therefore author’s opinion that a pre-determined diameter landmark overcomes the distortion resulting from different photograph angulations, both apico-coronal and mesio-distal, at the time of the software’s ruler calibration. Consequently, every measurement session has maintained the same calibration parameters not it was not influenced from deviations caused by free hand photograph frames.

A comparable method to obtain the same requisites (the same spatial plane at every evaluation session) would be the use of periodontal probe with a resin stent. However, this method could be of a higher cost and its applicability in the posterior area can be even more difficult.

Regarding the disadvantages, this technique might pose a difficulty in molars, as taking photos in this area is more challenging.

Ricci already proposed the measurement of soft tissue changes taking standardized photographs of study models that are positioned reproducibly with bite registration material [23]. Dental casts made in the course of therapy were mounted for photography one after each other on the same day, using the same registration compound. A grid was laid over the images, they were superimposed and reference lines were used to take measurements after importation into graphical software. The technique compared the outcome of a certain procedure with a linear error <0.1 mm. The method presented in the following study could be comparable to the procedure described by Ricci in terms of results and objectives.

It is inferable that similar outcomes have been found using the software associated with intraoral photographs combined with a pre-determined landmark for software calibration.

From the other hand, possible errors of the technique may be avoided with the elimination of cast models and with the bite registrations.

From the results of this work emerges that both inter and intra reading agreement of the method is high for its scientific and clinical applications; furthermore, the high level of inter operator concordance allows to compare data recorded by different clinicians in soft tissue monitoring; the range of discrepancies, higher for the inter-operator analysis, are probably caused by the different interpretation by the readers of the initial and end point of the measurement.

In fact the end point represented by the zenith of the gingiva could be different interpreted most of all for photographs of the posterior area which are, inevitably, not perpendicular.

A limit of this study is that it is not possible to confirm the absolute values amount of the measurements recorded because an alternative control method was not taken into account.

Other studies will be necessary to verify the reliability of distance measurements taken digitally on photographs using a known landmark on the tooth.

We can speculate that the present method can be extended also to natural teeth without provisional crowns using a known landmark, which could be positioned on the same plan of the tooth (for example a composite resin dot).

## CONCLUSION

The results obtained from the present analysis underlines that this method of calibration of well-known landmark with the software allows to obtain reproducible measurements in the comparison of gingival margin migration over the time. It’s therefore author’s opinion that it is valid, rapid and economic method for the monitoring of the soft tissue modifications for clinical and research applications.

Further studies will be necessary for the evaluation of the reliability of the distance measurements. This would enhance the comparability regarding the same technique performed in the different clinical trials.

## Figures and Tables

**Fig. (1a):**
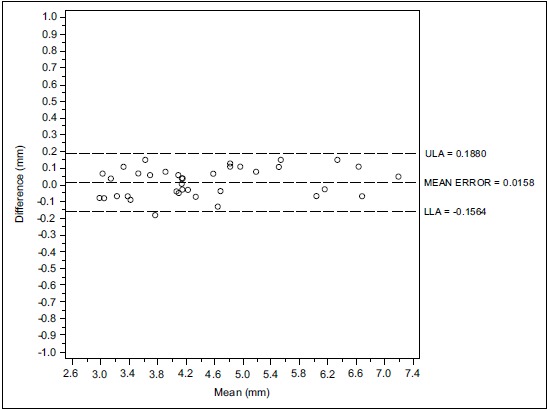
Intra operator concordance correlation Reader (D.F.).

**Fig. (1b):**
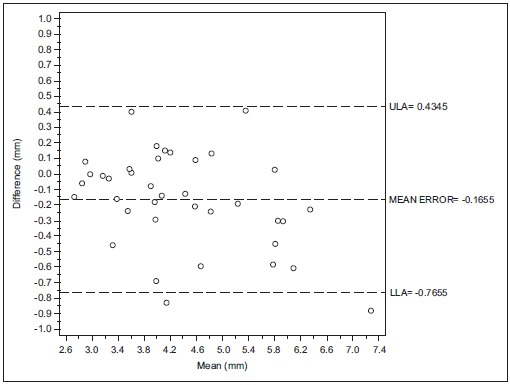
Intra operator concordance correlation Reader (F.L.).

**Fig. (1c):**
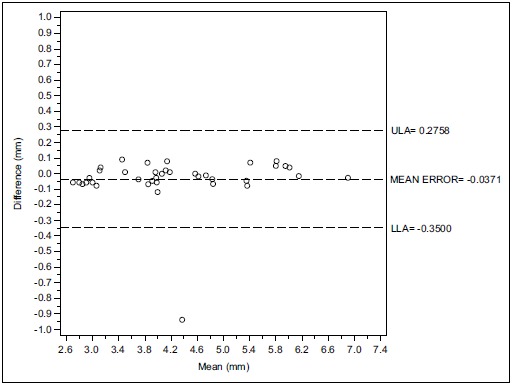
Intra operator concordance correlation Reader (F.A.).

**Fig. (2a):**
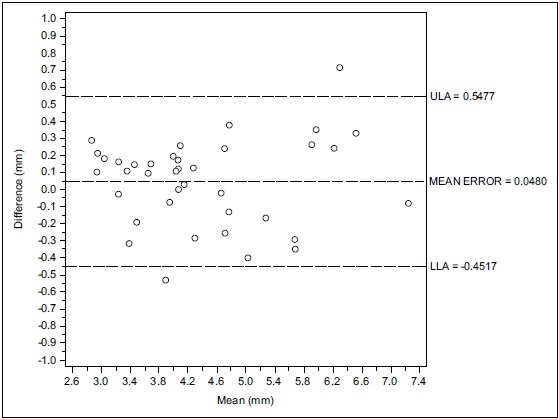
The inter-operator concordance between D.F. and L.I.

**Fig. (2b):**
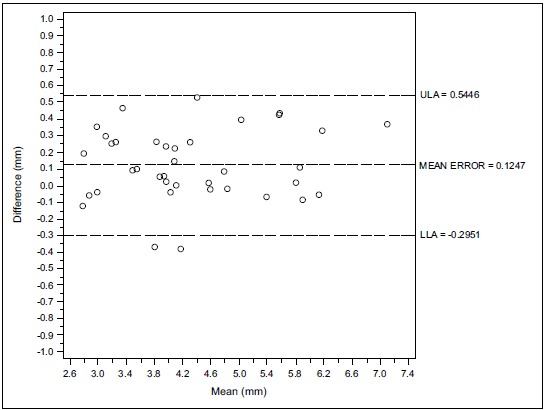
The inter-operator concordance between L.F. and F.A.

**Fig. (2c):**
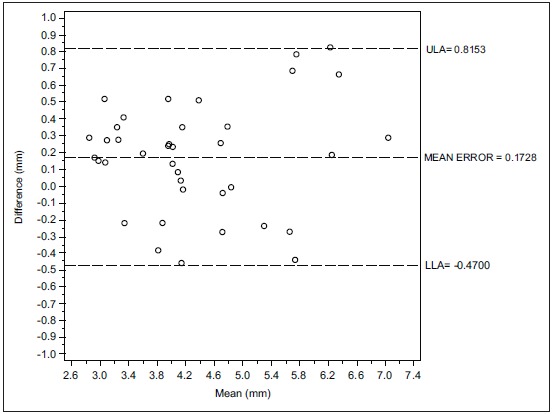
The inter-operator concordance between D.F. and F.A.

**Fig. (3):**
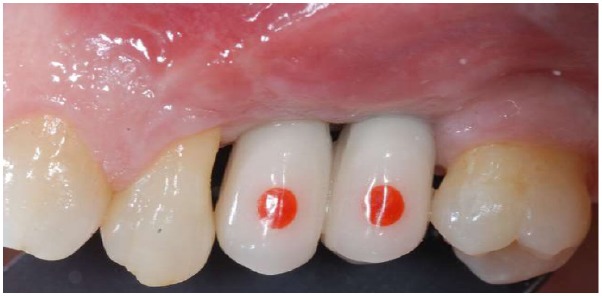
Circular landmark, centred in the middle of the tooth, with a previously determined diameter, on a provisional crown.

**Fig. (4):**
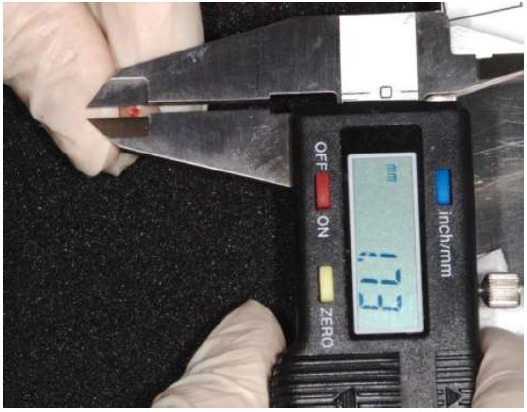
On-site evaluation of the landmark with a digital caliper.

**Fig. (5):**
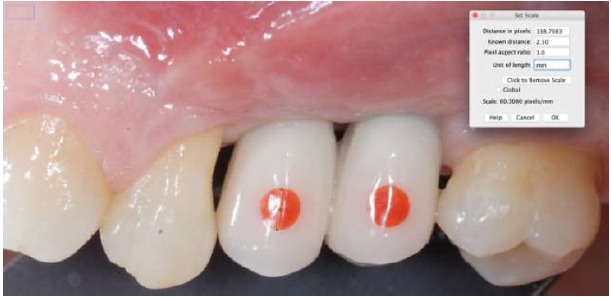
Calibration method of the centered landmark.

**Fig. (6):**
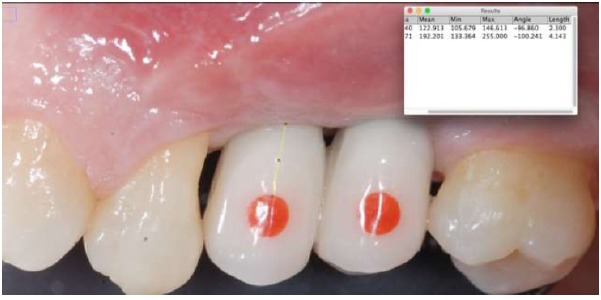
Distance measurement from the inferior border of the landmark to the free gingival margin.

**Table 1:** The data recorded from the three different examiners.

Examiner Names	Lower–upper IC 95% limits	Concordance
D.F.	0.995 – 0.998	0.996
F.L.	0.921 – 0.971	0.952
F.A.	0.934 – 0.999	0.988
